# From Biology to Clinical Practice: The Bone Marrow Microenvironment in Multiple Myeloma

**DOI:** 10.3390/jcm14020327

**Published:** 2025-01-08

**Authors:** Despina Fotiou, Eirini Katodritou

**Affiliations:** 1 Department of Clinical Therapeutics, National and Kapodistrian University of Athens, 11528 Athens, Greece; desfotiou@gmail.com; 2 Department of Hematology, Theagenion Cancer Hospital, 54639 Thessaloniki, Greece

## 1. Introduction

Multiple Myeloma (MM) is a complex hematological malignancy characterized by the clonal proliferation of malignant plasma cells within bone marrow (BM) [1]. The disease is also defined by the intricate interactions between these cells and their surrounding microenvironment, known as the bone marrow microenvironment (BMME) [2]. The BMME comprises various cellular components, including stromal cells, immune cells, and extracellular matrix (ECM) elements, which collectively create a supportive niche for myeloma cells [3,4]. This editorial aims to summarize and introduce the multifaceted role of the BMME in the pathogenesis and progression of MM, discuss current therapeutic strategies targeting the microenvironment, and identify gaps in knowledge that warrant further research.

### 1.1. The Role of the Bone Marrow Microenvironment in Multiple Myeloma

The BMME is a dynamic and interactive ecosystem that plays a significant role in regulating the behavior of clonal plasma cells. Key factors mediating this interaction include cytokines such as interleukin-6 (IL-6), B-cell activating factor (BAFF) and a proliferation-inducing ligand (APRIL), alongside other molecular mediators like Receptor activator of NF-kB (RANK) and Vascular Endothelial Growth Factor (VEGF). These factors, predominantly produced by bone marrow stromal cells (BMSCs), promote the survival, migration, and proliferation of myeloma cells [5,6]. IL-6, in particular, functions as a growth factor for immature plasma cells, but does not induce terminal differentiation, a process essential for sustaining the malignant phenotype [7,8]. Furthermore, the secretion of IL-6 by osteoclasts enhances the proliferation of myeloma cells but also promotes bone resorption through the upregulation of RANKL. This dual activity establishes a vicious cycle of bone destruction and tumor expansion [9,10,11].

In addition to cytokines, adhesion molecules play a critical role in the interaction between myeloma cells and the BMME. The expression of CD11b and LFA-1, along with the loss of CD56 and VLA-5, is associated with the migration of plasma cells from the bone marrow and the development of plasma cell leukemia [12]. Selectins also modulate the communication between plasma cells and neighboring stromal cells, further emphasizing the importance of adhesion in the pathophysiology of MM [13]. Adhesion molecules also seem to play a role in treatment response [14]. These interactions facilitate the survival of myeloma cells, and contribute to treatment resistance, as evidenced by the phenomenon of cell adhesion-mediated drug resistance (CAM-DR) [15].

The BMME is also characterized by the presence of various immune cells, including myeloid-derived suppressor cells (MDSCs) and regulatory T cells (Tregs), which create an immunosuppressive environment conducive to tumor growth. MDSCs exert their immunosuppressive effects through the production of inhibitory cytokines such as IL-10 and TGF-β, which inhibit the activation of effector T cells [16,17]. Additionally, MDSCs reduce tryptophan levels in the tumor microenvironment, impairing further T cell function [18,19]. Moreover, the infiltration of M2 macrophages within the BMME has been associated with a negative prognostic impact on treatment outcomes. These macrophages secrete pro-tumoral immunosuppressive agents such as IL-10 and TGF-β1, as well as pro-angiogenic factors like VEGF and FGF-2, which further promote an immunosuppressive environment and promote tumor progression [20]. This underscores the importance of targeting immune components within the BMME to improve therapeutic efficacy.

Regulatory T cells (Tregs), primarily identified as CD4+CD25+FoxP3+ cells, are increasingly recognized for their complex role in the immune regulation of multiple myeloma. They contribute to the maintenance of immune tolerance and the suppression of immune responses against tumor cells [21,22]. Studies have shown that Treg populations are often expanded in patients, particularly in the early stages of the disease, correlating with disease burden and paraprotein levels [23]. This increase in Tregs may reflect a compensatory mechanism to counteract the immune evasion strategies. Tregs have been shown to inhibit the proliferation of effector T cells and secrete immunosuppressive cytokines such as TGF-β and IL-10, which further modulate the immune response [22]. They have also been linked with reduced immune surveillance, contributing to disease progression.

The therapeutic implications of targeting Tregs in MM are being actively explored, as their immunosuppressive functions may hinder the efficacy of both conventional and novel immunotherapies. Strategies aimed at depleting or inhibiting Tregs, such as the use of monoclonal antibodies targeting specific surface markers like CTLA-4 and GITR, are under investigation [22], with the aim of enhancing antitumor immunity by restoring the function of effector T cells that are often suppressed in the presence of Tregs. Additionally, the modulation of Treg activity could potentially improve the efficacy of bortezomib- or lenalidomide-based therapy [23]. Overall, understanding the dual nature of Tregs in MM—both as regulators of immune tolerance and as potential barriers to effective therapy—remains a critical area of investigation in the quest to improve patient outcomes.

### 1.2. The Role of the Microenvironment in Drug Resistance

The BMME plays a crucial role in the development of drug resistance in MM. One of the primary mechanisms is the secretion of cytokines and growth factors that promote cell survival and proliferation. For example, IL-6 and IL-10, produced by BMSCs and Tregs, respectively, have been shown to enhance the survival of myeloma cells and protect them from the cytotoxic effects of chemoimmunotherapy [24]. Elevated IL-10 levels in MM patients correlate with lower response rates and poor prognosis, highlighting the importance of this cytokine in mediating drug resistance [25]. Additionally, the interaction between myeloma cells and the BMME leads to the activation of survival pathways that confer resistance to therapy. Clonal plasma cells can activate the AKT pathway through interactions with MSC-derived extracellular vesicles (EVs), which inhibit pro-apoptotic signaling pathways such as p38, p53, and c-Jun N-terminal kinase (JNK) [26,27]. This activation of survival pathways not only promotes cell proliferation, but also enhances resistance to various therapeutic agents, including proteasome inhibitors and immunomodulatory drugs [28]. The phenomenon of CAM-DR is particularly relevant in the context of drug resistance. Myeloma cells adhere to BMSCs through interactions involving adhesion molecules such as VLA-4 and CD44, which promote cell survival but also reduce the efficacy of chemotherapeutic agents [29]. This highlights the need for therapeutic strategies that disrupt these interactions to enhance treatment efficacy.

### 1.3. The Role of the Microenvironment in CAR-T and Bispecific Therapies

Recent advancements in cellular therapy, particularly CAR-T cell therapy and bispecific T-cell engagers (BiTEs), have shown great promise in the treatment of MM. CAR-T cell therapy involves the genetic modification of T cells to express chimeric antigen receptors (CARs) that specifically target antigens on myeloma cells, such as B-cell maturation antigen (BCMA) [30,31]. The BMME plays a significant role in the efficacy of CAR-T therapies, as the immunosuppressive microenvironment can hinder T-cell function and persistence. The BMME is characterized by the presence of Tregs and MDSCs, which can inhibit the activity of CAR-T cells [32]. The upregulation of co-inhibitory molecules, such as PD-1, or agonist molecules, such as T-cell immunoreceptors with immunoglobulin and ITIM domains (TIGIT) on activated T cells in the BMME can protect clonal plasma cells from immune attack [33,34]. Therefore, strategies aimed at modulating the BMME to enhance CAR-T cell efficacy are crucial. Ongoing research explores the use of immune checkpoint inhibitors or agonist proteins (such as TIGIT) in conjunction with CAR-T therapy to enhance T-cell activation and persistence [35,36]. Bispecific T-cell engagers (BiTEs) are another innovative therapeutic approach that harnesses the immune system to target myeloma cells. These agents are designed to simultaneously bind to CD3 on T cells and an antigen on myeloma cells, facilitating T-cell activation and cytotoxicity [37]. The efficacy of BiTEs is influenced to a significant extent by the BMME. Immunosuppressive cells the microenvironment can limit T-cell activation and function, thereby reducing the effectiveness of BiTEs [37]. Recent studies have indicated that the combination of BiTEs with agents that target the BMME, such as proteasome inhibitors, anti-CD38 monoclonal antibodies, or immune checkpoint inhibitors, may be able to enhance their therapeutic efficacy [38]. This approach aims to create a more favorable microenvironment for T-cell activity, ultimately improving treatment outcomes for patients with MM.

### 1.4. Understanding the Heterogeneity of the Bone Marrow Microenvironment

A critical aspect of the BMME is its heterogeneity, which can vary significantly among patients. This variability can influence response to treatment and long-term outcomes, underscoring the need for personalized therapeutic approaches. For instance, the expression of PD-L1, a key immune checkpoint molecule, has been associated with poor prognosis in relapsed/refractory MM [39]. While anti-PD-L1 therapies have shown promise in other malignancies, their efficacy in MM remains uncertain, as clinical studies have not demonstrated the anticipated benefits [40]. This highlights the importance of understanding the unique characteristics of the BMME in individual patients with MM and to adapt the treatment approach to effectively target both clonal plasma cells and their supportive microenvironment.

Moreover, the role of Bregs, a small B-cell subset with immunosuppressive properties, in mediating the evasion of myeloma plasma cells from the immune system is an emerging area of research. Bregs promote an immunosuppressive microenvironment through the production of IL-10 and the activation of the APRIL/TACI axis, which supports the survival of malignant plasma cells [41]. Understanding the mechanisms underlying Breg functions and their interactions with myeloma cells may provide valuable insights into novel therapeutic strategies aimed at reversing immune suppression in MM. Recent studies have highlighted the importance of subclone-specific cellular interactions within the BMME. For example, CD44 on myeloma cells interacts with LGALS9 on BMME cells, which could be validated by emerging spatially resolved techniques [42,43]. To identify new therapeutic targets, we need to understand the resistance mechanisms that occur secondary to subclone-specific interactions.

### 1.5. Gaps in Knowledge and Future Directions

Despite significant advancements in our understanding of the BMME’s role in MM, several gaps remain that necessitate further investigation. A deeper understanding of the molecular mechanisms governing the interactions between myeloma cells and various components of the BMME is required. The roles of IL-6 and other cytokines are well-documented, but the specific signaling pathways activated in response to these factors remain to be fully elucidated. Another active area of research is the impact of hypoxia within the BMME on myeloma cell behavior and treatment resistance, particularly given the aberrant expression of hypoxia-inducible factors by myeloma cells [15,44].

The BMME also needs to be better characterized at the level of the individual. The complexity of the BMME, influenced by factors such as genetic abnormalities and patient-specific immune profiles, suggests that a one-size-fits-all approach may be inadequate. We also need to identify biomarkers that can predict treatment response and resistance. A personalized approach would enhance the efficacy of existing therapies and allow for novel therapeutic strategies. The combinatory targeting of multiple components of the BMME is another approach that could potentially overcome resistance and immune evasion and improve outcomes.

## 2. Conclusions

The bone marrow microenvironment plays a pivotal role in the pathogenesis and progression of multiple myeloma, influencing tumor growth, survival, and therapeutic resistance. Understanding the intricate interactions within the BMME has significant implications for clinical practice, particularly in the development of novel therapeutic strategies. However, critical gaps in knowledge remain, particularly regarding the molecular mechanisms governing these interactions and the heterogeneity of the BMME among patients. Addressing these gaps through focused research will be essential for advancing the treatment of multiple myeloma and improving patient outcomes.
